# Safety and effects of scorpion-derived antimicrobial peptides as an alternative to antibiotic growth promoters in broilers: growth performance, immune function, and intestinal development

**DOI:** 10.3389/fvets.2025.1677663

**Published:** 2025-10-09

**Authors:** Mingyang Gao, Zhengli Wang, Hongfeng Zhao, Zihui Li, Hong Shen

**Affiliations:** College of Animal Science and Technology, Shihezi University, Shihezi, China

**Keywords:** feed additives, antimicrobial peptides, broilers, production performance, amino acid composition, gut health promotors

## Abstract

Antimicrobial peptides (AMPs) derived from scorpion venom have emerged as promising environmentally sustainable feed additives due to their biosafety and ability to metabolize into natural amino acids without residues. This study investigates IsCT, a cytotoxic peptide from *Isometroides scorpions*, for its potential application in yellow-feathered broiler production. The study began with *in vitro* antimicrobial susceptibility testing against major livestock pathogens (*Staphylococcus aureus* ATCC 6538, *Salmonella typhimurium* ATCC 14028, *Escherichia coli* ATCC 25922, and *Streptococcus agalactiae* ATCC 13813), followed by biosafety evaluations using chicken erythrocytes and Kunming mice. A feeding trial with 360 broilers assigned birds to six dietary treatments: basal diet control, IsCT supplementation at 25, 50, 100, or 200 mg/kg, and a ciprofloxacin control (50 mg/kg). IsCT exhibited concentration-dependent antibacterial activity with no hemolytic effects and demonstrated biosafety in murine models. During days 1–21 and 22–42, IsCT supplementation significantly improved feed conversion efficiency, carcass quality, immunoglobulin levels, and intestinal development in broilers. IsCT shows broad-spectrum efficacy and growth-promoting potential, supporting its use as a sustainable feed additive in yellow-feathered broiler production systems.

## Graphical abstract

IsCT functions as a broad-spectrum antimicrobial peptide and effective growth/immune enhancer in poultry production.

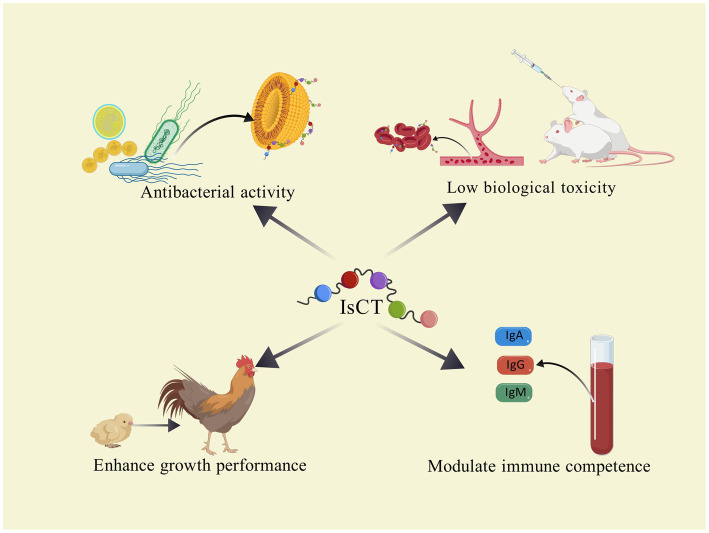

## 1 Introduction

Since the discovery of penicillin, Antibiotic Growth Promoters (AGPs) have become integral to poultry farming for their remarkable efficacy in disease prevention and growth enhancement. Their utilization significantly contributed to the intensification of livestock production by reducing morbidity and mortality rates while improving feed conversion ratios. However, the extensive and often indiscriminate application of AGPs has led to the emergence and dissemination of antimicrobial resistance (AMR), a pressing public health concern that compromises the efficacy of therapeutic antibiotics in both animals and humans (1–3). In response to these challenges, global regulatory agencies have implemented stricter policies regarding antibiotic use in animal feeds, driving the quest for sustainable and effective alternatives. Amid this transition, green bacteriostatic agents—derived from natural sources and characterized by favorable environmental profiles—have become the focus in the development of new feed additives (4–9). Among these, antimicrobial peptides (AMPs) represent a highly promising category. These short peptides, typically consisting of 12–50 amino acids, are produced by a wide range of organisms as a first line of defense against pathogens. AMPs possess multiple biological properties, including broad-spectrum inhibitory effects against bacteria, fungi, viruses, and even parasites. Unlike conventional antibiotics, which often target specific molecular pathways, AMPs generally exert their effects through non-specific mechanisms, such as disrupting microbial membranes, modulating immune responses, and inhibiting biofilm formation. This mode of action significantly reduces the likelihood of resistance development, making AMPs an attractive option for long-term use in animal production (7).

Isalo scorpion cytotoxic peptide (IsCT) is a short-chain antimicrobial peptide originally isolated from the venom of the *Israeli scorpion Leiurus quinquestriatus*. It is widely recognized as one of the shortest naturally occurring antimicrobial peptides identified to date, with a simple yet effective sequence (ILGKIWEGIKSLF-NH₂) (10, 11). This compact molecular structure contributes not only to its potent and broad-spectrum antimicrobial properties but also to its strong resistance to proteolytic degradation (10, 12), enhancing its stability in biological environments. Beyond its direct antimicrobial effects, research has shown that IsCT exhibits low cytotoxicity toward mammalian cells, which underscores its potential suitability for therapeutic and agricultural applications (10, 12). Moreover, IsCT has been found to modulate and enhance the host’s innate immune response, further increasing its utility as an immunostimulatory agent. In the context of animal production, IsCT demonstrates significant promise as a sustainable alternative to conventional antibiotics. It effectively inhibits the growth of major pathogenic bacteria commonly found in livestock settings, thereby reducing the incidence of infectious diseases and improving overall animal health. Additionally, dietary supplementation with IsCT has been linked to enhanced growth performance and improved antioxidant capacity in animals, contributing to more efficient and environmentally friendly farming practices (13, 14).

The application of innovative feed additives like IsCT is especially relevant for yellow-feathered broilers—a characteristic Asian poultry species with large-scale farming operations and significant economic importance—that face inherent challenges: intensive high-density farming enhances productivity but compromises growth performance and increases disease susceptibility. Although traditional antibiotic additives like ciprofloxacin effectively promote production, their long-term use intensifies antimicrobial resistance, hepatorenal toxicity risks, and drug residue concerns, driving industry demand for sustainable alternatives. Against this backdrop, the scorpion venom peptide IsCT emerges as a promising candidate due to its multiple advantages. It has potent antibacterial activity, immunomodulatory functions, high biosafety (metabolizing into residue-free natural amino acids), and environmental compatibility (15). Nevertheless, despite commanding 25% of Southeast Asia’s poultry market owing to superior sensory attributes, yellow-feathered broilers remain critically understudied compared to extensively researched white-feathered breeds—particularly regarding novel antimicrobial peptides like IsCT. This research gap is especially notable given the fundamental physiological differences between these breeds: unlike white-feathered broilers, which are bred for extremely rapid growth and high feed efficiency, yellow-feathered broilers exhibit a slower growth rate, superior carcass quality, and distinct metabolic and nutrient partitioning mechanisms. These breed-specific characteristics highlight the need for tailored nutritional and health interventions, necessitating systematic exploration of IsCT’s growth-promoting effects following validation of its pathogen-specific antibacterial efficacy and safety profile in this under-characterized breed. AMPs at 0–100 mg/kg have demonstrated growth-promoting and immunomodulatory effects in broilers. Gao et al. (16) found maggot-derived AMPs (100–300 mg/kg) improved growth/immunity in yellow-feathered broilers. Notably, although IsCT has demonstrated considerable potential in aquaculture—for example, by improving intestinal barrier function and development in grass carp (*Ctenopharyngodon idella*) (17) and enhancing growth and intestinal immunity in fish—studies evaluating its effects on monogastric animals, especially poultry, remain limited. Therefore, the present study was designed to address this unmet need. We hypothesized that dietary supplementation with IsCT would dose-dependently improve growth performance, enhance immune responses, and promote intestinal development in yellow-feathered broilers, yielding effects comparable to or better than the conventional antibiotic ciprofloxacin, without adversely affecting health parameters. The present study aimed to evaluate the effects of IsCT supplementation at 25, 50, 100, and 200 mg/kg on growth and immunological parameters in yellow-feathered broilers, using a ciprofloxacin control (50 mg/kg) (18, 19) as a benchmark. It should be specifically noted that although antibiotics such as ciprofloxacin were historically used as growth-promoting additives, their use is now largely restricted due to regulatory changes. In this study, ciprofloxacin was included as a positive control to benchmark the efficacy of IsCT against a known antimicrobial agent. By comparing the antibacterial efficacy, hemolytic activity, and overall impact on broiler performance between IsCT and a commonly used antibiotic, this work seeks to provide a comprehensive assessment of IsCT’s potential as a feed additive in poultry production.

## 2 Materials and methods

### 2.1 Ethical approval

All experimental procedures involving animals were conducted in accordance with institutional animal welfare guidelines. All procedures involved in this study were formally reviewed and approved by the Shihezi University Animal Ethics Committee (Approval No: A2025-546, A2025-545). The experiment was carried out at the Animal Center of Shihezi University (Xinjiang, China), where extensive facilities and resources were utilized to ensure the precision and reliability of the research endeavors. This study strictly adhered to internationally recognized animal welfare and ethical standards throughout its duration. All experimental animals were housed in clean and spacious environments with continuous access to sufficient food and water. Prior to euthanasia, mice and broiler chickens were administered 3% (v/v) isoflurane inhalation anesthesia via an induction chamber until loss of consciousness, followed by humane euthanasia via cervical dislocation. All procedures were performed following the core principle of minimizing animal suffering, thereby reducing stress and distress to the greatest extent feasible.

### 2.2 Antimicrobial peptide and reagents

IsCT was purchased from Shanghai Sunshile Company (Shanghai, China) and was synthesized via solid-phase synthesis with >95% purity. Melittin was obtained from PeptideGen Biotechnology Co., Ltd. (Hangzhou, China) at 98% purity. *Staphylococcus aureus* ATCC 6538, *Salmonella typhimurium* ATCC 14028, *Escherichia coli* ATCC 25922, and *Streptococcus agalactiae* ATCC 13813 were provided by the Microbiology Laboratory of the College of Animal Science and Technology at Shihezi University. Mouse Enzyme-linked immunosorbent assay (ELISA) kits were acquired from Jiangsu Jingmei Biological Technology Co., Ltd. (Jiangsu, China). Fully automated biochemical analyzer (Model: BK1200, Shandong Biobase Biotechnology Co., Ltd., China). Light microscope (Model: BX53, Olympus Corporation, Tokyo, Japan). Full-spectrum microplate reader (Model: Multiskan SkyHigh, Thermo Fisher Scientific, United States).

### 2.3 Experimental animals and housing conditions

Based on studies by Zhu et al. and our laboratory’s prior experience with comparable models, 60 Kunming (KM) mice were utilized in the present investigation. These 5-week-old, SPF (Specific Pathogen Free) grade mice, with an average body weight of 29.82 ± 1.5 g, were all sourced from the Experimental Animal Center of Xinjiang Medical University. All mice were housed in a strictly controlled SPF environment, in which optimal conditions were maintained: temperature at 22 ± 2 °C, relative humidity at 55–60%, and a 12-h light/dark cycle. This ensured consistent health status of the experimental animals and the reliability of the results.

Yellow-feathered broilers were sourced from Shihezi Sansan Hatchery. At the Animal Station of Shihezi University. The sample size was determined based on common practices in poultry nutrition research (20–23) and our laboratory’s previous experience with similar models. A total of 360 healthy, vaccinated chickens with similar body weight (initial body weight: 29.0 ± 0.5 g) were selected. Birds were housed in three-tier vertical broiler cages at 0.2 m^2^/bird density under 24-h incandescent lighting (20 lux) with mechanical ventilation. Environmental conditions were maintained at 60–70% relative humidity (hygrometer-monitored) and controlled temperature: 35 ± 0.5 °C for Week 1, reduced by 3 °C weekly until stabilizing at 22 ± 1 °C from Day 28. The temperature-humidity index (THI), a key indicator of thermal comfort and heat stress in poultry, was calculated daily based on the recorded temperature and humidity using the following formula established by Yan et al. (24). The daily THI profile throughout the experimental period is presented in Supplementary Table S1 and  Graphical abstract. Feed and water were provided ad libitum via nipple drinkers and trough feeders. Biosecurity measures include daily manure removal and weekly iodophor disinfection (at a 1:200 dilution).

### 2.4 Antibacterial activity assay

The Oxford cup assay was performed according to Xiang’s method (25). In brief, *Staphylococcus aureus*, *Salmonella* spp., *Escherichia coli*, and *Streptococcus agalactiae* were resuscitated according to the method described by Zeng et al. (26), and the suspensions were prepared to a concentration of 1 × 10^8^ CFU/mL. A 100 μL aliquot of each bacterial suspension was spread onto agar plates. Four Oxford cups were placed on each plate, followed by the injection of IsCT solutions at concentrations of 1 mg/mL and 0.5 mg/mL to assess antibacterial activity against the four pathogens. Inhibition zone diameters were recorded, with sterilized water as the negative control and 0.2 mg/mL ciprofloxacin as the positive control. All measurements were performed in three independent replicate experiments, and the mean values were calculated. Three independent biological replicates were performed for each bacterium, with each replicate using a fresh bacterial culture prepared on a different day. The mean values were calculated from these biological replicates.

### 2.5 Determination of minimum inhibitory concentration

MIC was determined using the microbroth dilution method. Following bacterial culture, strains were diluted to 1 × 105 CFU/mL. In 96-well microplates, 50 μL of serially diluted IsCT solutions were added to 50 μL of bacterial inoculum, yielding final peptide concentrations of 16, 32, 64, 128, 256, 512, and 1,024 μg/mL. After 24-h incubation at 37 °C, absorbance at OD600 was measured using a microplate reader. All measurements were performed in three independent replicate experiments, and the mean values were calculated. Three independent biological replicates were performed for each bacterium, with each replicate using a fresh bacterial culture prepared on a different day. The mean values were calculated from these biological replicates.

### 2.6 Hemolytic activity assay

Chicken blood was centrifuged (1,000 × g, 10 min, 4 °C) to isolate erythrocytes. Cells were washed thrice with equal-volume Phosphate-Buffered Saline (PBS) and were centrifuged after each wash. Serial IsCT and Melittin dilutions (final concentrations: 0.25–4 mg/mL) were mixed with equal volumes of erythrocyte suspension. Controls included untreated cells (negative) and 0.1% Triton X-100-treated cells (positive). Following 1 h incubation (37 °C), samples were microscopically examined (1,000×). Remaining solutions were centrifuged; supernatant absorbance (OD₅₇₀) was measured. Minimum hemolytic concentration (MHC) was defined as the peptide concentration causing 10% hemolysis. All measurements were performed in three independent replicate experiments, and the mean values were calculated. For the hemolysis assay, blood samples from six chickens were used as biological replicates, with each sample tested in triplicate.

### 2.7 *In vivo* toxicity test of IsCT

After 7 days of acclimation, KM mice were randomly divided into 5 groups based on body weight:0.2 mL sterile saline was administered to the CON group via intragastric gavage; 0.2 mL of 2.5 mg/mL IsCT peptide solution was administered to Group I; 0.2 mL of 5 mg/mL IsCT peptide solution was administered to Group II; 0.2 mL of 10 mg/mL IsCT peptide solution was administered to Group III; and 0.2 mL of 20 mg/mL IsCT peptide solution was administered to Group IV. Each group comprised 6 replicates with 2 mice per replicate. After 14 consecutive days of administration, one mouse per replicate was selected for sample collection on Day 15. The health status of the mice was assessed and scored according to the criteria established by Zhu et al. (9). Briefly, the scoring system was defined as follows: 5 = normal activity, 4 = hunched posture with reduced mobility (but ambulatory), 3 = hypokinesia and lacrimation, 2 = moribund, and 1 = dead. Organ indices and serum biochemical parameters were subsequently analyzed.

### 2.8 Broiler farming experimental design and diets

This study utilized a randomized complete block design (RCBD), with initial body weight as the blocking factor. Briefly, a total of 360 healthy yellow-feathered broilers were first ranked by their initial body weight and then assigned to one of six treatment groups, each consisting of 10 replicates, ensuring that birds within each replicate had similar body weights: CON (basal diet), I (basal diet + 25 mg/kg IsCT), II (basal diet + 50 mg/kg IsCT), III (basal diet + 100 mg/kg IsCT), IV (basal diet + 200 mg/kg IsCT), and CIP (basal diet + 50 mg/kg ciprofloxacin). Diets were formulated according to Chinese Nutrient Requirements for Yellow-Feathered Broilers (NY/T 33–2004), with complete formulations detailed in Table 1. Vaccination against Newcastle disease virus (NDV) and infectious bronchitis virus (IBV) was administered at Days 7 and 21. Growth metrics (ADG: Average Daily Gain, ADFI: Average Daily Feed Intake, F/G: Feed-to-Gain Ratio) were recorded weekly. On days 22 and 43 of the trial, one bird per replicate within each treatment group, selected based on proximity to the mean body weight (resulting in six birds per group, serving as biological replicates), was humanely euthanized via cervical dislocation. Liver, bursa of Fabricius, and serum samples were subsequently collected and snap-frozen in liquid nitrogen for further analysis.

**Table 1:** Composition and nutrient levels of the basal diet (air-dry basis) %.

Item	Content	
	1–21 days	22–42 days
Ingredients		
Corn	55.00	67.30
Soybean meal	32.20	20.50
Bran	2.50	2.50
Fish meal	2.00	2.50
Soya-bean Oil	3.30	2.00
Premix^1^	5.00	5.00
NaCl	0	0.2
Total	100.00	100.00
Nutrient levels^2^		
ME(MJ/kg)	12.24	14.98
CP	21.54	17.92
Ca	0.88	0.87
TP	0.80	0.82
AP	0.40	0.41
Lys	1.12	0.97
Met	0.49	0.46
Thr	0.80	0.66

1 The premix provides per kilogram of feed: VA 180000 IU, VD 70000 IU, VE 450 IU, VK 30 mg, 70 mg, 60 mg, niacin 600 mg, calci-um pantothenate 260 mg, biotin 1.7 mg, folic acid 17 mg, Fe 10,000 mg, Cu 350 mg, Mn 1,500 mg, Zn 2000 mg, Ca 14 mg, P 6 mg, NaCl 7 mg, methionine3 mg.

2 ME, amino acid and AP were calculated by referring to the “Chinese Feed Composition and Nutritional Value Table (29th Edition 2018).” CP, Ca and TP were, respectively, referred to GB/T 6432, GB/T 6436 and GB/T 6437.

### 2.9 Growth performance measurement

Body weight (BW), average daily feed intake (ADFI), average daily gain (ADG), and feed-to- gain ratio (F/G) were calculated for days 1–21 and days 22–42 phases as follows: broilers underwent a 12-h fast (feed withdrawal with ad libitum water access) prior to weighing on days s 21 and 42 using calibrated digital scales (±0.1 g); BW was recorded individually at d1/d21/d42; The ADFI, ADG, F/G were calculated according to the following standardized formulas:


$$\begin{array}{l} \mathrm{ADG}\;(g/\text{bird}/\mathrm{day})=(\text{Final}\;\mathrm{BW}-\text{Initial}\;\mathrm{BW})/\\ \text{days in phase} \end{array}$$

$$\begin{array}{l} \text{ADFI}\;(g/\text{bird}/\mathrm{day})=\text{Cumulative feed consumption}/\\ \left(\text{birds}\times \text{days}\right) \end{array}$$

F/G=ADFI/ADG

All measurements were performed in three independent replicate experiments, and the mean values were calculated.

### 2.10 Carcass quality

On days 21 and 42 of the trial, broilers were euthanized, followed by feather removal and exsanguination. Carcass weight and composition—including dressing percentage, breast muscle yield, thigh muscle yield, and abdominal fat percentage—were measured according to the method described by Namted et al. (27).

### 2.11 Blood biochemical analysis

Serum biochemical parameters were measured according to the method described by Liu et al. (28). Chicken blood was collected using vacuum tubes. The serum was separated by centrifugation at 3000 × g for 15 min at 4 °C. Serum levels of total protein (TP), albumin (ALB), globulin (GLB), as well as the activities of aspartate aminotransferase (AST), glucose (GLU), and alanine aminotransferase (ALT) in 21-day-old and 42-day-old broiler chickens were measured using a fully automated biochemical analyzer. All measurements were performed in three independent replicate experiments, and the mean values were calculated.

### 2.12 Immune organ index calculation

The immune organ indices were measured according to the method described by Ma et al. (29). Simply put, spleens, thymuses, and bursae of Fabricius were rinsed with sterile physiological saline and weighed. The immune organ indices were calculated according to the following standardized formulas:


$$\begin{array}{l} \text{Bursa index}\;(\mathrm{mg}/\mathrm{kg})=\text{Bursa weight}\;(g)/\\ \text{Body weight}\;(\mathrm{kg}) \end{array}$$

$$\begin{array}{l} \text{Spleen index}\;(\mathrm{mg}/\mathrm{kg})=\text{Spleen weight}\;(g)/\\ \text{Body weight}\;(\mathrm{kg}) \end{array}$$

$$\begin{array}{l} \text{Thymus index}\;(\mathrm{mg}/\mathrm{kg})=\text{Thymus weight}\;(g)/\\ \text{Body weight}\;(\mathrm{kg}) \end{array}$$

All measurements were performed in three independent replicate experiments, and the mean values were calculated.

### 2.13 Immune parameter assay

Serum immune parameters were determined according to the method described by Liu et al. (30). Briefly, serum concentrations of immunoglobulin A (IgA), immunoglobulin G (IgG), and immunoglobulin M (IgM) were quantified according to the manufacturer’s instructions. Briefly, chicken blood samples were processed as described in Section 2.10, mixed with corresponding reagents, and incubated at 37 °C for 30 min. After five washes, the enzyme conjugate was added and reacted at 37 °C for 30 min. Following another five washes, chromogenic substrate was added and developed at 37 °C for 10 min. Finally, a stop solution was added, and absorbance was measured at 450 nm using a microplate reader. All measurements were performed in three independent replicate experiments, and mean values were calculated.

### 2.14 Observation of intestinal morphology

Morphological analysis of intestinal tissue was performed according to the method described by Khan et al. (31). Briefly, intestinal tissue segments were immersed in 4% paraformaldehyde (PFA) solution for 24 h under light-protected fixation, and then were subjected to paraffin embedding and sectioning at 3 μm thickness. Histological evaluation was performed using hematoxylin and eosin (H&E) staining. Villus height and crypt depth were measured under an optical microscope, with subsequent calculation of the villus-to-crypt ratio.

### 2.15 Statistical analysis

Statistical analysis was performed using SPSS 27.0 (IBM Corp., Armonk, NY, USA). Normality of continuous data (e.g., body weight, villus height, immunoglobulin concentrations) was assessed using the Shapiro–Wilk test. Homogeneity of variances was confirmed with Levene’s test, followed by one-way analysis of variance (One-way ANOVA, for comparing differences among the six dietary treatment groups) and *post hoc* LSD tests (for pairwise comparisons between groups, e.g., CON vs. Group III) where appropriate. Data visualization (e.g., bar graphs for carcass quality, line graphs for hemolytic activity) was conducted using GraphPad Prism 10.1 (GraphPad Software, San Diego, CA, United States). Pearson correlation analysis (to assess linear relationships between continuous variables, e.g., villus-to-crypt ratio and serum IgG levels) and hierarchical clustering were performed using Origin 2021 (OriginLab Corp., Northampton, MA, United States). The clustering analysis employed hierarchical clustering with complete linkage (to group treatment groups with similar growth/immune/gut development patterns). Statistical significance was uniformly defined as *p* < 0.05 (significant difference) and *p* < 0.01 (highly significant difference) for all analyses (including ANOVA, LSD pairwise comparisons, and Pearson correlation). Investigators remained blinded to group assignments during data collection and analysis to minimize subjective bias in data recording and statistical interpretation.

## 3 Results

### 3.1 Inhibitory zone diameters

**Figure 1:**
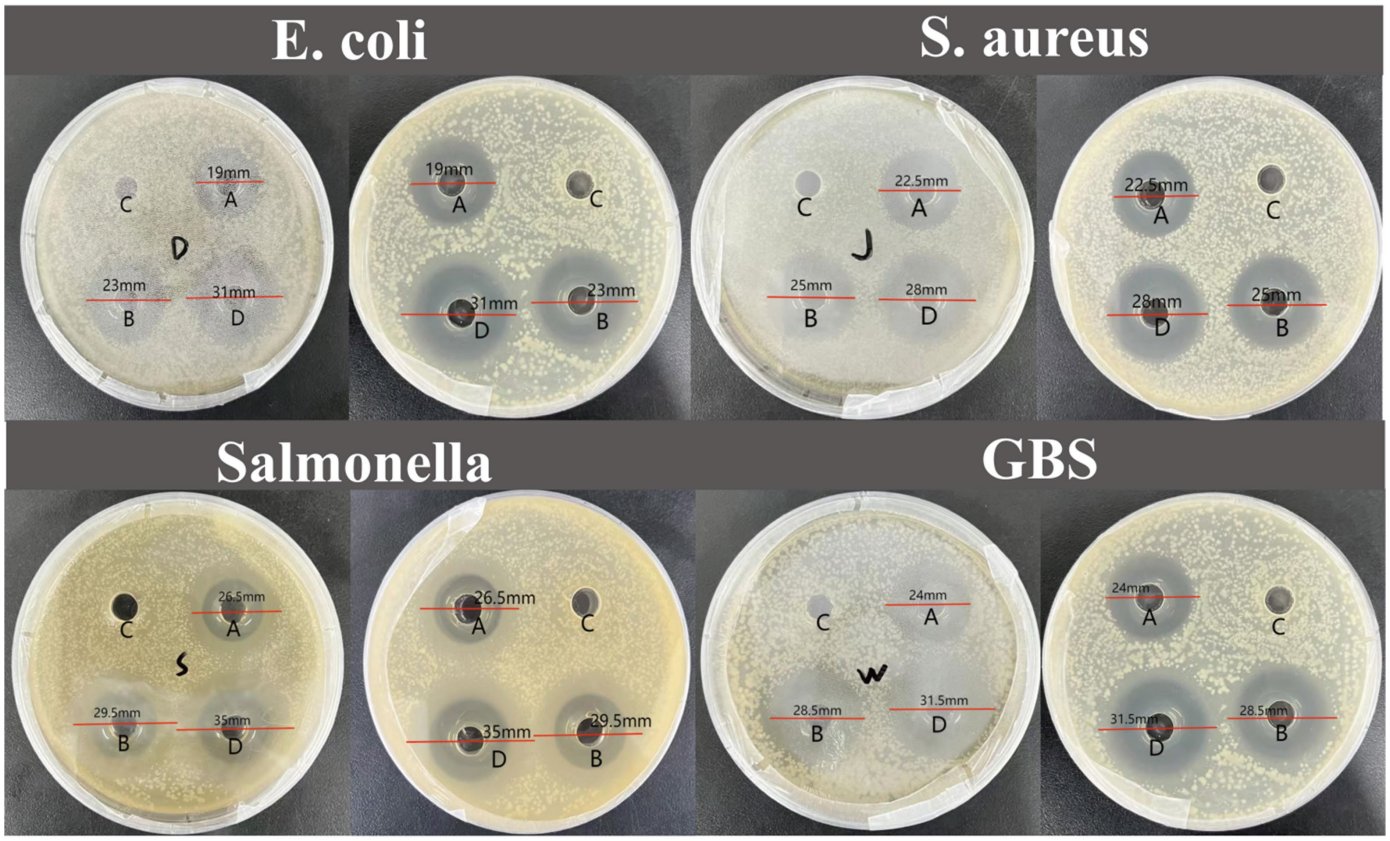
Inhibition zone diameters for the four bacterial species tested. (A) 0.5 mg/mL IsCT exhibited inhibition zone diameters of 19 mm against *E. coli,* 22.5 mm against *S. aureus*, 26.5 mm against Salmonella, and 24 mm against GBS; (B) 1 mg/mL IsCT exhibited inhibition zone diameters of 23 mm against *E. coli*, 25 mm against *S. aureus*, 29.5 mm against *Salmonella*, and 28.5 mm against GBS; (C) sterile water exhibited no antibacterial activity; (D) 0.2 mg/mL ciprofloxacin exhibited inhibition zone diameters of 31 mm against *E. coli*, 28 mm against *S. aureus,*35 mm against Salmonella, and 31.5 mm against GBS.

The experimental results indicate that IsCT exhibits bacteriostatic ability against all four tested bacterial strains. Specifically, at 0.5 mg/mL, IsCT exhibits an inhibitory zone of 19 mm against *Escherichia coli*. Furthermore, at both tested concentrations, IsCT produces inhibitory zones larger than 20 mm against all strains (Figure 1). At 0.5 mg/mL, IsCT exhibits significantly higher bacteriostatic ability against *Salmonella* than that against *Escherichia coli* and *Staphylococcus aureus* (*p* = 0.012, *p* = 0.023). Its bacteriostatic ability against *Staphylococcus aureus and GBS* was significantly higher than that against *Escherichia coli* (*p* = 0.026, *p* = 0.019). The bacteriostatic ability of 1 mg/mL IsCT against GBS and *Salmonella* was significantly higher than that against *Escherichia coli* (*p* = 0.021, *p* = 0.035). Bacteriostatic zones are not observed in the control group (Table 2).

**Table 2:** Inhibitory zone diameters of two concentrations of IsCT against four bacteria.

Item	The diameter of the bacteriostatic ring (mm)				SEM	*p*-value
	*S. aureus*	Salmonella	*E. coli*	GBS		
0.5 mg/mL IsCT	22.50^b^	26.50^a^	19.00^c^	24.00^ab^	0.876	<0.001
1 mg/mL IsCT	25.00	29.50^a^	23.00^b^	28.50^a^	0.577	0.007
0.2 mg/mL Ciprofloxacin	28.00^c^	35.00^a^	31.00^b^	31.50^b^	0.927	<0.001

The results are expressed as means and mean standard errors (SEM). In the same row, data labeled with different lowercase letters indicate significant differences (*p* < 0.05), data labeled with different uppercase letters indicate highly significant differences (*p* < 0.01), while data labeled with the same letters or no letters indicate no significant differences (*p* > 0.05).

### 3.2 Mic

As shows in Table 3, IsCT demonstrates MIC values of 32 μg/mL against both *E. coli* and GBS, while exhibiting an MICs of 64 μg/mL against *Salmonella* and 128 μg/mL against *S. aureus* In comparison, ciprofloxacin shows consistent MICs of 1 μg/mL against *E. coli*, *S. aureus*, and *Salmonella*, with an MIC of 4 μg/mL against GBS.

**Table 3:** Minimum inhibitory concentration (MIC) of IsCT.

Item	*S. aureus*	Salmonella	*E. coli*	GBS
IsCT	128 μg/mL	64 μg/mL	32 μg/mL	32 μg/mL
Ciprofloxacin	1 μg/mL	1 μg/mL	1 μg/mL	4 μg/mL

### 3.3 Hemolytic activity

**Figure 2:**
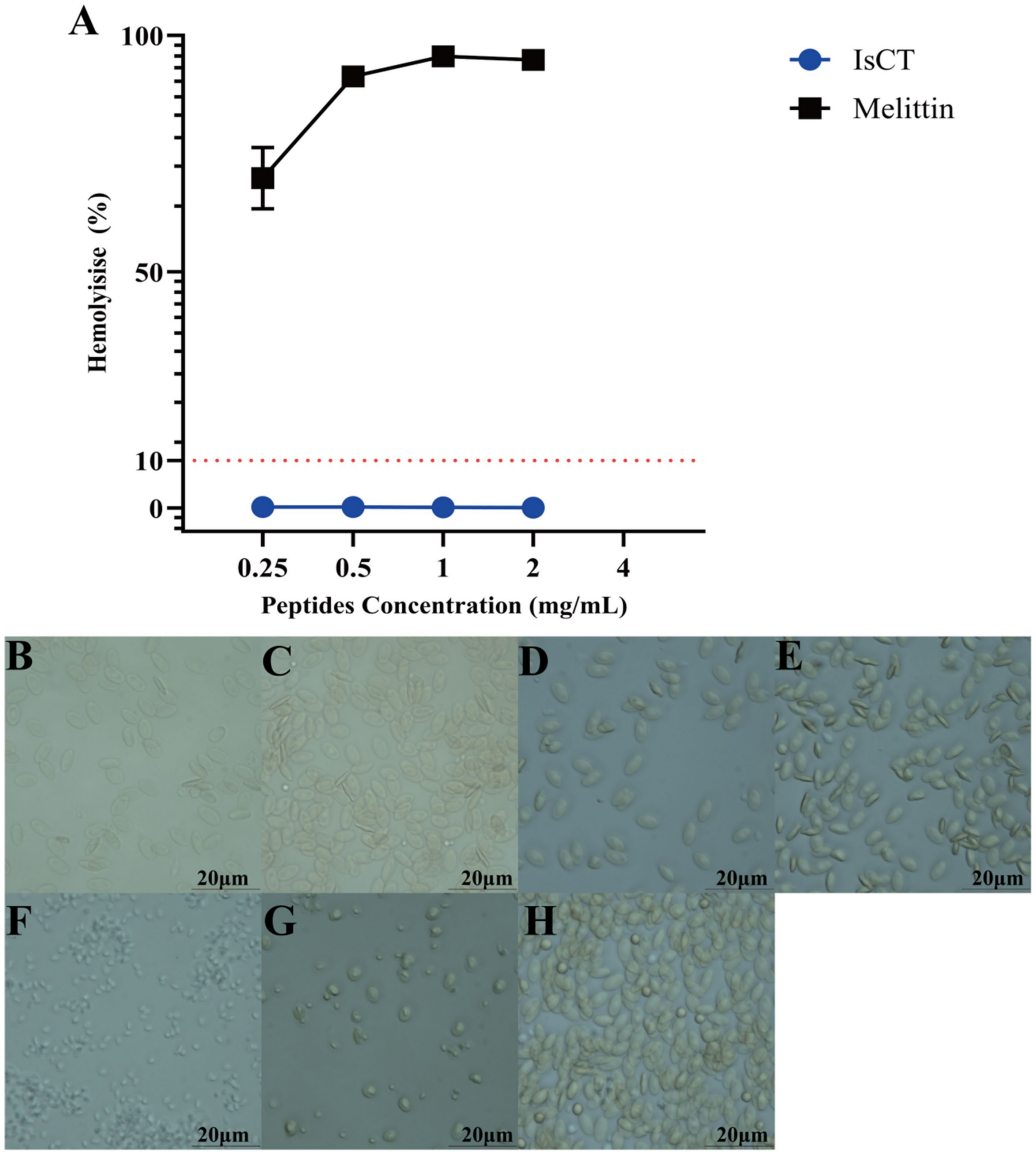
IsCT has low hemolytic toxicity. **(A)** Concentration-dependent hemolytic activity of the antimicrobial peptide IsCT (blue line) and the reference peptide melittin (black line). **(B–H)** Bright-field microscopy images of chicken erythrocytes after treatment for 1 h at 37 °C: **(B)** 0.25 mg/mL IsCT, **(C)** 0.5 mg/mL IsCT, **(D)** 1 mg/mL IsCT, **(E)** 2 mg/mL IsCT, **(F)** 4 mg/mL IsCT, **(G)** 1 mg/mL Triton X-100 (positive control for complete lysis), **(H)** Untreated group (negative control). Scale bar: 20 μm.

**Figure 3:**
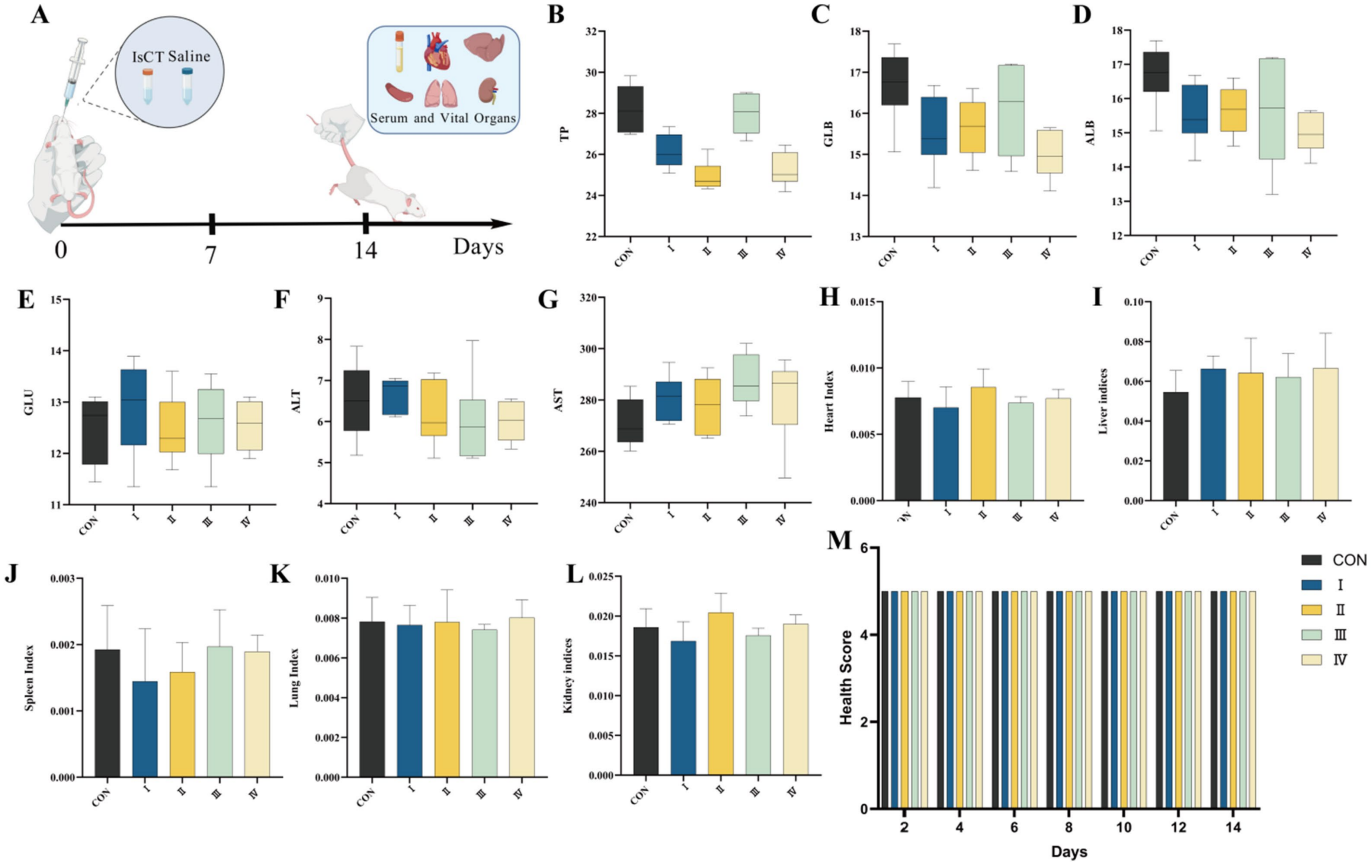
Dietary supplementation with IsCT demonstrates favorable *in vivo* safety profiles in a mouse model. **(A)** Schematic of the experimental design for the in vivo toxicity assessment of IsCT in mice. Serum biochemical parameters: **(B)** total protein (TP), **(C)** globulin (GLB), **(D)** albumin (ALB), **(E)** glucose (GLU), **(F)** alanine aminotransferase (ALT), **(G)** aspartate aminotransferase (AST). Organ indices: **(H)** heart index, **(I)** liver index, **(J)** spleen index, **(K)** lung index, **(L)** kidney index. **(M)** Health score of mice following IsCT administration.

As depicted in Figure 2, IsCT shows a hemolysis rate close to 0% at 2 mg/mL but detectable hemolytic activity at 4 mg/mL. In contrast, Melittin at the same concentrations induces nearly complete hemolysis (≈100%). Figure 3 displays representative micrographs of treated erythrocytes. Chicken erythrocytes exposed to lower IsCT concentrations (0.25–2 mg/mL) maintain structural integrity with cell volumes comparable to untreated controls, indicating absence of hemolysis. Conversely, those treated with 4 mg/mL IsCT show significantly reduced volumes—though still larger than cells lysed by 0.1% Triton X-100—accompanied by morphological transitions from elliptical to spherical configurations. Partial cellular fragmentation into irregular shapes and reduced intracellular content are observed, confirming progressive hemolysis.

### 3.4 *In vivo* toxicity

As shown in Figure 3, after 14 days of gavage administration, all mice remained active with no visible abnormalities. Compared to the control group, there are no significant changes in body weight, vital organ indices, or serum biochemical parameters in the gavage groups.

### 3.5 Growth performance

As presented in Table 4, at 21 days of age, the final body weight of the CIP group increases by 19 and 21% compared to the CON group and Group II, respectively (*p* = 0.006, *p* = 0.007). During the 21–42 days period, the final body weight of Group III increases by 9, 13, 20, and 11% compared to the CON group, Group I, Group II, and Group IV, respectively (*p* = 0.004, p = 0.006, *p* = 0.002, *p* = 0.004). The average daily gain (ADG) increases by 22, 19, and 15% compared to the CON group, Group I, and Group II, respectively (*p* = 0.005, *p* = 0.013, *p* = 0.028). The feed-to-gain ratio (F/G) decreases by 5% compared to the CON group (*p* = 0.037). The final body weight of the CIP group increases by 14 and 8% compared to the CON group and Group II, respectively (*p* = 0.023, *p* = 0.039). The ADG increases by 15% compared to the CON group (*p* = 0.047).

**Table 4:** The effect of IsCT on the growth performance.

Item	Group						SEM	*p*-value
	CON	I	II	III	IV	CIP		
BW (g) at 1 day of age	30.09	30.02	29.98	30.89	30.07	29.81	0.146	0.365
BW (g) at 21 day of age	423.30^b^	449.19	412.39^Bb^	460.35	477.54	502.73^Aa^	8.597	0.007
BW (g) at 42 day of age	1126.25^Bc^	1197.71^Bbc^	1137.50^Bc^	1363.67^Aa^	1229.91^bc^	1284.63^ab^	20.441	<0.001
1–21 days								
ADFI/g	37.25	36.82	36.77	38.55	37.91	40.28	0.496	0.311
ADG/g	18.73	19.96	18.21	20.53	21.31	22.52	0.619	0.366
F/G	2.03	1.87	2.07	1.92	1.80	1.79	0.059	0.700
21–42 days								
ADFI/g	90.84	89.34	92.31	90.26	88.73	95.24	0.842	0.255
ADG/g	33.48^Bc^	34.52^bc^	35.64^bc^	40.99^Aa^	36.65	38.43^ab^	0.792	0.051
F/G	2.75^a^	2.60	2.60	2.22^b^	2.43	2.50	0.06	0.144

The results are expressed as means and mean standard errors (SEM). In the same row, data labeled with different lowercase letters indicate significant differences (*p* < 0.05), data labeled with different uppercase letters indicate highly significant differences (*p* < 0.01), while data labeled with the same letters or no letters indicate no significant differences (*p* > 0.05).

### 3.6 Carcass quality

**Figure 4:**
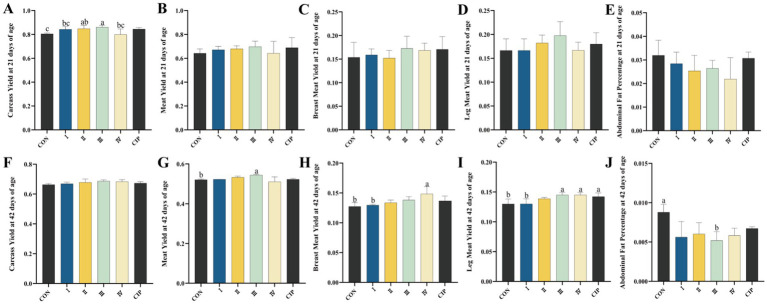
Dietary supplementation with IsCT improves carcass traits in broilers. **(A–E)** Measurements at 21 days of age: **(A)** carcass yield, **(B)** meat yield, **(C)** breast muscle yield, **(D)** thigh muscle yield, **(E)** abdominal fat percentage. **(F–J)** Measurements at 42 days of age: **(F)** carcass yield, **(G)** meat yield, **(H)** breast muscle yield, **(I)** thigh muscle yield, **(J)** abdominal fat percentage. Different lowercase letters indicate statistically significant differences (*p* < 0.05).

As shown in Figure 4, at 21 days of age, the carcass yield of Group II increases by 5% compared to the CON group (*p* = 0.043). The carcass yield of Group III increases by 7, 8, and 8% compared to the CON group, Group I, and Group IV, respectively (*p* = 0.021, *p* = 0.031, *p* = 0.039). At 42 days of age, the meat yield of Group III increases by 4% compared to the CON group (*p* = 0.022), while the abdominal fat percentage decreases by 41% compared to the CON group (*p* = 0.014). The breast muscle yield of Group IV increases by 17 and 15% compared to the CON group and Group I, respectively (*p* = 0.027, *p* = 0.025). The thigh muscle yield of Groups III, IV, and the CIP group increases by 12, 12, and 9% compared to the CON group, and by 12, 12, and 9% compared to Group I, respectively (*p* = 0.01, *p* = 0.01, *p* = 0.028, *p* = 0.01, *p* = 0.01, *p* = 0.029).

### 3.7 Blood biochemical parameters

**Figure 5:**
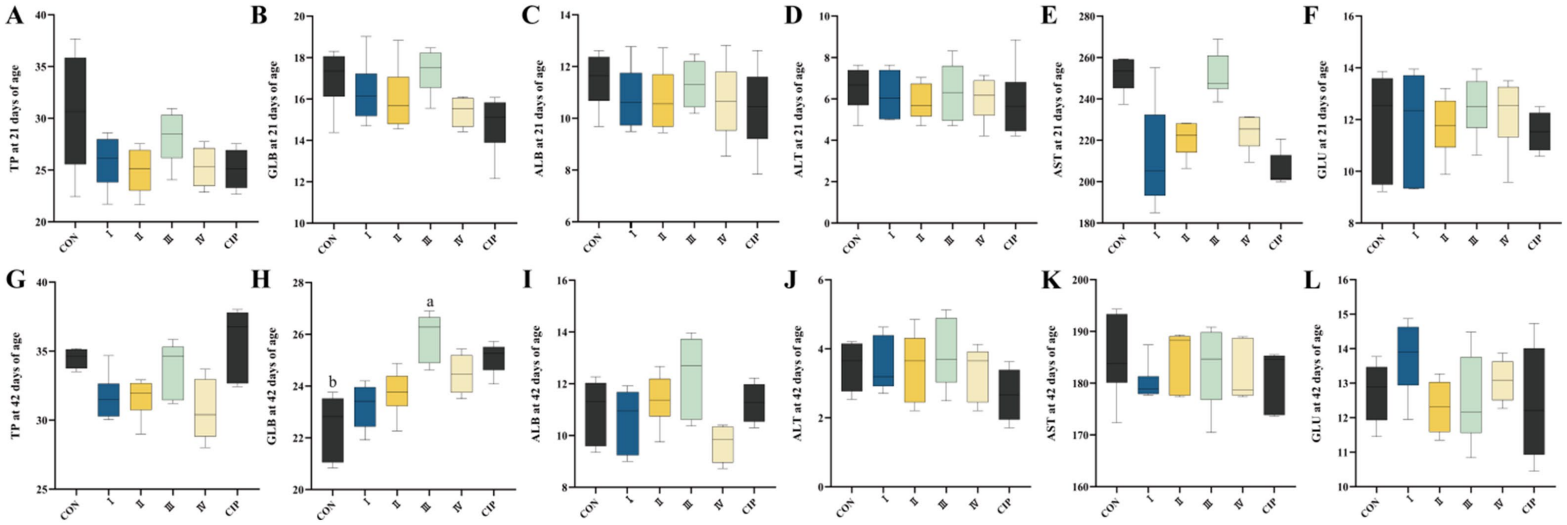
Dietary supplementation with IsCT exhibits relatively minor effects on the serum biochemical profile of broilers overall. **(A–F)** Parameters measured at 21 days of age: **(A)** total protein (TP), **(B)** globulin (GLB), **(C)** albumin (ALB), **(D)** alanine aminotransferase (ALT), **(E)** aspartate aminotransferase (AST), **(F)** glucose (GLU). **(G–L)** Parameters measured at 42 days of age: **(G)** TP, **(H)** GLB, **(I)** ALB, **(J)** ALT, **(K)** AST, **(L)** GLU. Different lowercase letters indicate statistically significant differences (*p* < 0.05).

As shown in Figure 5, dietary supplementation with IsCT or antibiotics has no significant effect on serum biochemical parameters of yellow-feathered broilers during 1–21 days of age. In contrast, during 21–42 days of age, compared to the CON group, the serum GLB level in Group III increases by 17% (*p* = 0.021).

### 3.8 Immune organ indices

**Figure 6:**
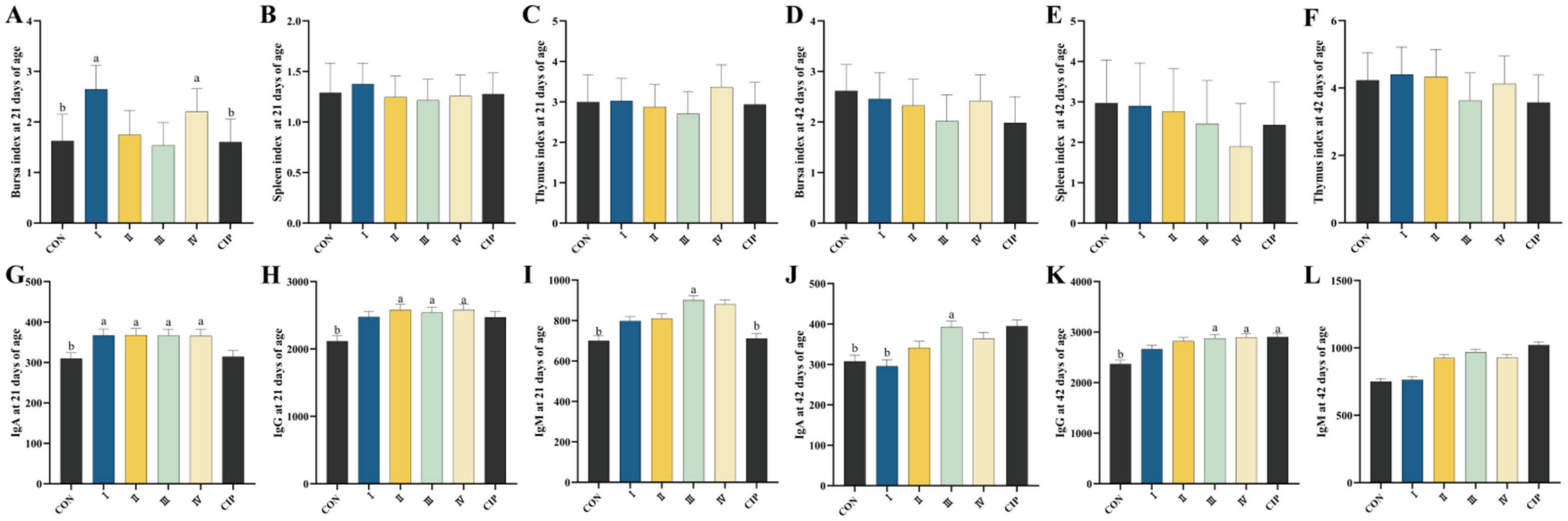
Dietary supplementation with IsCT enhances immune function in broilers. **(A–C)** Immune organ indices at 21 days of age: **(A)** thymus index, **(B)** spleen index, **(C)** bursa of Fabricius index. **(D–F)** Immune organ indices at 42 days of age: **(D)** thymus index, **(E)** spleen index, **(F)** bursa of Fabricius index. **(G–I)** Serum immunoglobulin levels at 21 days of age: **(G)** immunoglobulin A (IgA), **(H)** immunoglobulin G (IgG), **(I)** immunoglobulin M (IgM). **(J–L)** Serum immunoglobulin levels at 42 days of age: **(J)** IgA, **(K)** IgG, **(L)** IgM. Different lowercase letters indicate statistically significant differences (*p* < 0.05).

As shown in Figures 6A–F, during 1–21 days of age, The Bursa index of Group I and Group IV increased by 62 and 26%, and by 65 and 38%, respectively, compared to the control group and the CIP group (*p* = 0.001, *p* = 0.043, p = 0.001, *p* = 0.036) However, dietary supplementation with IsCT or ciprofloxacin has no significant effect on immune organ indices of yellow-feathered broilers during 21–42 days of age.

### 3.9 Serum immune parameters

As presented in Figures 6G–L, at 21 days of age, serum IgA levels in Groups I–IV increase by 19, 19, 18, and 18%, respectively, compared to the CON group (*p* = 0.036, *p* = 0.032, *p* = 0.035, *p* = 0.032). Serum IgA levels in Groups II–IV increase by 22, 20, and 22%, respectively, compared to the CON group (*p* = 0.023, *p* = 0.025, *p* = 0.012). The serum IgM level in Group III increases by 28% compared to the CON group (*p* = 0.019). At 42 days of age, serum IgA in Group III increases by 27 and 32% compared to the CON group and Group I, respectively (*p* = 0.016, *p* = 0.022). Serum IgG levels in Group III, Group IV, and the CIP group all increase by 22% compared to the CON group (*p* = 0.035, *p* = 0.025, *p* = 0.031).

### 3.10 Intestinal morphology

**Figure 7:**
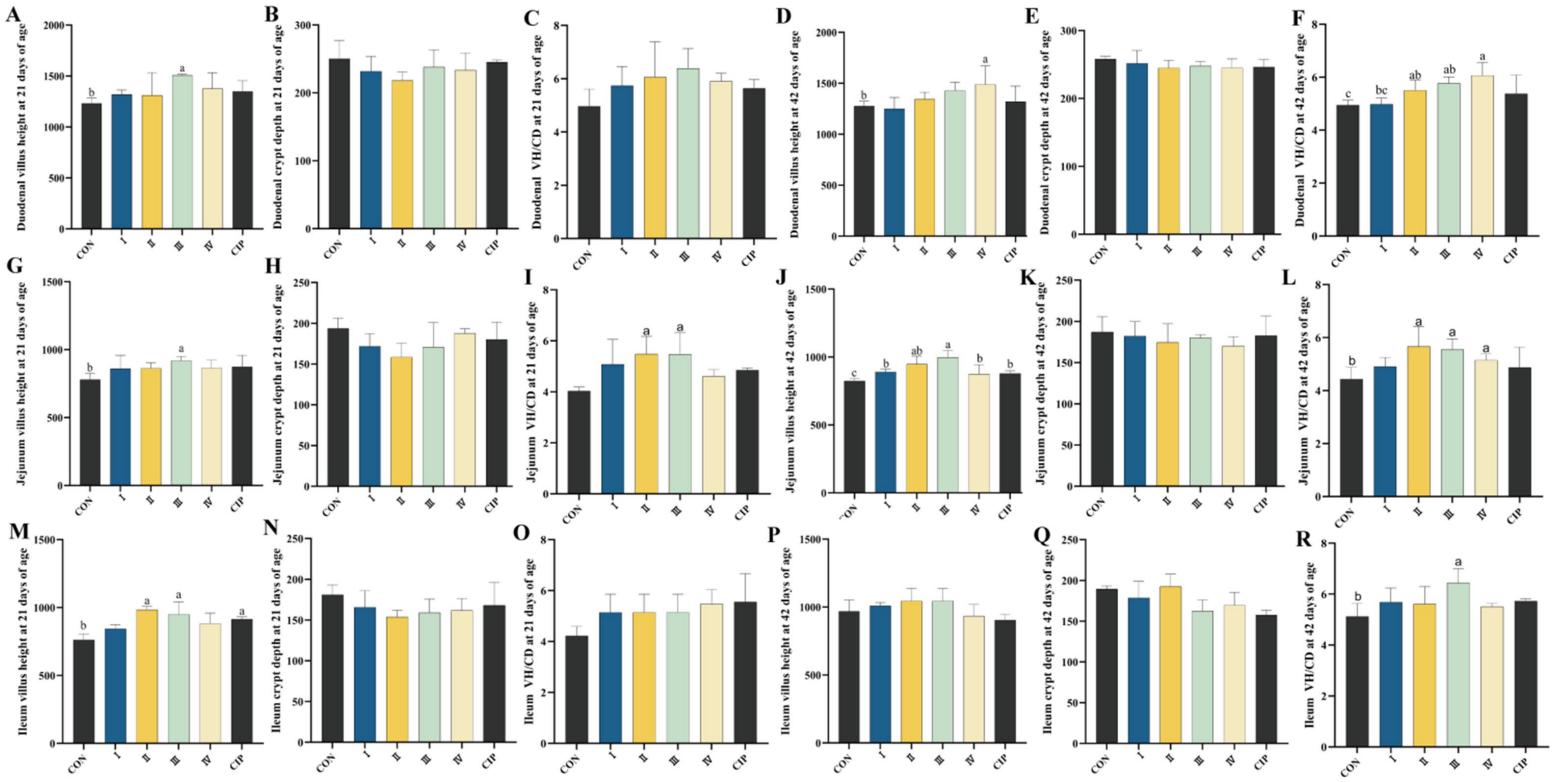
Dietary supplementation with IsCT improves intestinal morphology in broilers. **(A–C)** Duodenal morphology at 21 days of age: **(A)** villus height (VH), **(B)** crypt depth **(CD)**, **(C)** villus height to crypt depth ratio (VH/CD). **(D–F)** Duodenal morphology at 42 days of age: **(D)** VH, **(E)** CD, **(F)** VH/CD. **(G–I)** Jejunal morphology at 21 days of age: **(G)** VH, **(H)** CD, **(I)** VH/CD. **(J–L)** Jejunal morphology at 42 days of age: **(J)** VH, **(K)** CD, **(L)** VH/CD. **(M–O)** Ileal morphology at 21 days of age: **(M)** VH, **(N)** CD, **(O)** VH/CD. **(P–R)** Ileal morphology at 42 days of age: **(P)** VH, **(Q)** CD, **(R)** VH/CD. Different lowercase letters above bars indicate significant differences (*p* < 0.05).

As shown in Figure 7, compared to the control group (CON), Group I shows an 8% increase in jejunal villus height at 42 days (*p* = 0.044). Group II exhibits a 13% increase in ileal villus height at 21 days and a 7% improvement in the duodenal villus-to-crypt ratio at 42 days (*p* = 0.016, *p* = 0.020). Group III demonstrates increases in villus height of 18, 12, and 21% in the duodenum, jejunum, and ileum, respectively, at 21 days (*p* = 0.026, *p* = 0.031, *p* = 0.012), along with a 19% increase in jejunal villus length at 42 days (*p* = 0.019). Additionally, the jejunal villus-to-crypt ratio improves at 21 days, and further increases of 13, 17, and 20% are observed across the three intestinal segments at 42 days (*p* = 0.033, *p* = 0.018, *p* = 0.020, *p* = 0.035). Group IV shows a 10% increase in duodenal villus length and a 23% increase in the jejunal villus-to-crypt ratio at 42 days (*p* = 0.024, *p* = 0.015). The CIP group exhibits significant improvements in ileal villus length at 21 days and in jejunal villus length at 42 days (*p* = 0.027, *p* = 0.039).

### 3.11 Correlation analysis

**Figure 8:**
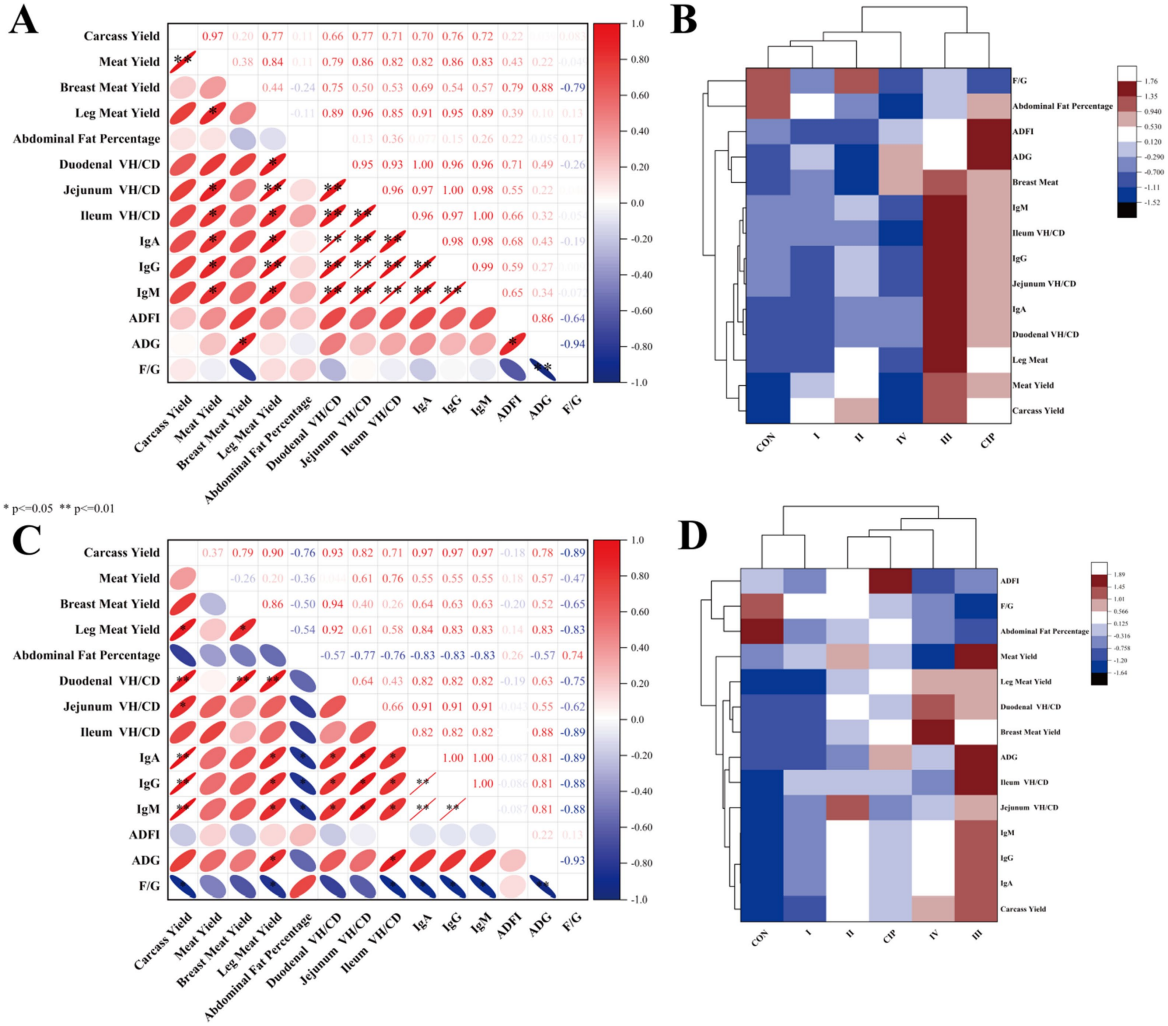
Dietary supplementation with IsCT induces strong correlations among growth performance, immune function, and gut development in broilers. **(A)** Correlation heatmap of growth performance, immune parameters, and intestinal development indices in 21-day-old broilers. **(B)** Cluster analysis of growth performance, immune parameters, and intestinal development indices in 21-day-old broilers. **(C)** Correlation heatmap of growth performance, immune parameters, and intestinal development indices in 42-day-old broilers. **(D)** Cluster analysis of growth performance, immune parameters, and intestinal development indices in 42-day-old broilers. Red signifies positive correlation, with intensity proportional to depth (deeper red indicating stronger positive correlation); blue denotes negative correlation, with intensity similarly proportional to depth (deeper blue indicating stronger negative correlation). *p < 0.05, **p < 0.01.

Figures 8,C show correlations among growth performance, immunity, and gut development at 21 and 42 days. At 21 days, carcass yield, thigh muscle yield, immunoglobulin levels, and villus-to-crypt ratio are positively correlated (*r* > 0.7, *p* < 0.05) and negatively correlated with FCR and abdominal fat percentage (*r* < −0.7, *p* < 0.05). At 42 days, meat production rate and thigh muscle yield are positively correlated with immunoglobulin levels and villus-to-crypt ratio (*r* > 0.7, *p* < 0.05). Immunoglobulin levels and villus-to-crypt ratio are highly significantly correlated (*r* > 0.7, *p* < 0.01). Figures 8B,D compare overall growth, immune, and gut development levels across groups at 21 and 42 days. Both IsCT and ciprofloxacin improve all measured indicators, with 100 mg/kg IsCT showing the most consistent and significant enhancements.

## 4 Discussion

### 4.1 IsCT *in vitro* antimicrobial activity

Gram-positive bacteria (e.g., *Staphylococcus aureus*, *Streptococcus agalactiae*) and Gram-negative bacteria (e.g., *Salmonella* spp., *Escherichia coli*) are prevalent in animal husbandry (32, 33), causing clinical conditions including pneumonia, mastitis, meningitis, septicemia, and diarrhea (3, 34–36). These diseases exhibit high mortality rates with complex transmission pathways that elude comprehensive control, threatening sustainable livestock production. This study demonstrates IsCT’s strongest inhibitory effects against GBS and *Salmonella*, followed by *S. aureus*, with weaker activity against *E. coli*. The variation in sensitivity among bacterial species may be attributed to differences in cell envelope structure. Gram-negative bacteria possess an outer membrane rich in lipopolysaccharides, which may hinder peptide penetration, whereas Gram-positive bacteria have a thick peptidoglycan layer that might be more susceptible to membrane-active peptides like IsCT. This aligns with literature showing insect-derived AMPs have broad-spectrum activity against these pathogens. For example, Pereira et al. (37) reports Melittin MIC = 4 μg/mL against *E. coli*; Picoli et al. (38) documents 6–7 μg/mL against *S. aureus*; Jiang et al. (39) determined cecropin P1 MIC = 4 μg/mL against *Salmonella* and *E. coli*; Denardi et al. (40) showed cecropin A activity against *Salmonella* at 2 μg/mL. These findings align with our observations. As hydrophobic amino acids, Ile and Leu enhance the peptide’s hydrophobicity (mean hydrophobic index > 2.1), facilitating its insertion into bacterial lipid bilayers (41, 42). This action disrupts the outer membrane of Gram-negative bacteria and the peptidoglycan layer of Gram-positive bacteria. This disruption induces leakage of intracellular contents, thereby potentiating IsCT’s antibacterial efficacy.

In this study, we employ Oxford cup and microbroth dilution methods to assess IsCT’s antibacterial effects against four bacterial strains. Both methods confirm IsCT’s strongest inhibition against *Streptococcus agalactiae* and Salmonella, followed by *S. aureus*, with weaker effects against *E. coli*. However, Oxford cup results occasionally show variability. This may relate to IsCT’s amino acid composition: its sequence is rich in hydrophobic residues, particularly C-terminal Ile (isoleucine). Additionally, N-terminal Phe (phenylalanine) acts as a hydrogen donor, promoting hydrogen bonds between the N-terminal amino group and Phe’s main-chain carbonyl group, thereby affecting spatial conformation. Trp (tryptophan) in the mid-to-C-terminal region contributes to these structural features, potentially causing peptide chain aggregation (43, 44). The microbroth dilution method likely reflects IsCT’s true antibacterial activity more accurately due to refined drug concentration gradients. Conversely, high IsCT concentrations in Oxford cup assays may induce peptide aggregation (45), impairing agar diffusion and consequently affecting inhibition zone formation and measurement.

### 4.2 Safety profile of IsCT

The membrane-lytic bactericidal mechanism unique to antimicrobial peptides may induce hemolytic toxicity (46–48), necessitating careful therapeutic index evaluation for clinical translation (49). Approximately 70% of AMPs exhibit hemolytic activity (50), making it a critical safety indicator and major translational hurdle (46). Our findings demonstrate that IsCT shows no observable hemolysis at concentrations effective against all four target pathogens. Furthermore, despite belonging to the *α*-helical peptide class, IsCT demonstrates significantly lower hemolytic activity than Melittin. This divergence stems from altered target specificity: strong hydrophobicity enables preferential interaction with eukaryotic plasma membranes. Liu et al. (51) show that reducing Melittin’s hydrophobicity significantly diminishes hemolysis while maintaining antibacterial efficacy, confirming hydrophobicity’s role in AMP selectivity. IsCT, LL-37, and other low-hydrophobicity peptides exhibit enhanced binding to negatively charged bacterial membranes, achieving superior biosafety (52).

Following the verification of IsCT’s *in vitro* erythrocyte toxicity, assessment of its *in vivo* toxicity was essential prior to evaluating its effects on broiler growth performance. In serum analysis, total protein and albumin levels provide an overview of hepatic and renal function, while elevated AST and ALT activities serve as critical indicators of liver damage. As demonstrated by Zhu et al. (9), administration of the antimicrobial peptide IK3 induces no significant alterations in these parameters in mice. In our 14-day gavage study, mice treated with various doses of IsCT exhibit no significant changes in body weight, abnormal behaviors, or alterations in relative weights of vital organs. Subsequent biochemical analyses further confirm its safety. These findings are consistent with results from the broiler feeding trial, where only broilers continuously fed 100 mg/kg IsCT until 42 days of age show elevated globulin levels. Based on subsequent experimental data, we infer this change results from increased serum immunoglobulin (IgA, IgG, and IgM) concentrations.

### 4.3 Effects of IsCT on growth performance

Growth performance is a key determinant of livestock economic value (53). This study shows dietary supplementation with IsCT at 100 mg/kg significantly improved average daily gain (ADG) and feed efficiency (F/G) in broilers during the 22–42 day period without affecting feed intake (ADFI). This suggests that IsCT enhances nutrient utilization rather than appetite stimulation. Tai et al. (54) found 3% recombinant piscidin EP increases body weight and reduces F/G; Choi et al. (55) report 60 mg/kg AMP-A3 improves growth performance and nutrient retention—aligning with our ADG increase under equivalent feed intake, confirming conserved nutrient partitioning mechanisms. Carcass yield, breast meat yield, thigh meat yield, and abdominal fat percentage are critical metrics for evaluating poultry production efficiency. These measurements provide clear insights into slaughter performance, muscle development, and fat distribution. As growth-promoting bioactive compounds, AMPs have demonstrated potential to enhance carcass traits in livestock. Shi et al. (56) report that dietary supplementation with composite AMPs increases carcass weight and meat yield in beef cattle by modulating ruminal microbiota and metabolism. However, research on AMP effects on broiler carcass characteristics remains limited. Our study addresses this knowledge gap by revealing that dietary IsCT significantly increases breast meat yield and thigh meat yield while reducing abdominal fat percentage in 42-day-old broilers. These findings carry notable practical implications, demonstrating IsCT’s capacity to optimize body composition by promoting deposition of commercially valuable muscle cuts and reducing undesirable abdominal fat accumulation. The potential factor contributing to the reduced abdominal fat percentage in broilers by IsCT may be its impact on key biomolecules involved in adipose tissue formation, such as the activation of the peroxisome proliferator-activated receptor (PPAR) signaling pathway. Activation of the PPARγ signaling pathway can disrupt the endocrine system and lead to adipose tissue accumulation (57). Szychowski et al. (58) demonstrated that short-chain peptides influence the expression of *Pparγ* and *β*-galactosidase (β-Gal) in cellular assays, suggesting a possible mechanism by which IsCT, as a peptide-based substance, reduces abdominal fat deposition in broilers.

Notably, IsCT’s effects were more pronounced in the grower phase than in the starter phase. This may be due to higher protein turnover and muscle accretion rates in older birds, where improved nutrient absorption and metabolic efficiency have greater impact (59, 60). Although IsCT contains several essential amino acids—such as leucine, which modulates feed intake and hypothalamic NPY/AgRP expression (61); isoleucine, associated with muscle protein synthesis and microbial homeostasis (62–64); lysine, which supports intestinal development, improves feed utilization, and optimizes amino acid balance (65); and tryptophan, involved in GLP-1 and bile acid signaling that may reduce the feed-to-gain ratio (F/G) (66, 67)—it is important to note that at the low inclusion level of 100 mg/kg, any direct nutritional contribution from these amino acids is negligible. Instead, these structural components may facilitate functional activities such as membrane interaction and receptor signaling. The greater efficacy observed during days 22–42, a phase characterized by substantial muscle development, may reflect enhanced protein metabolism mediated through such functional pathways. Furthermore, IsCT may influence broiler physiology through multiple functional mechanisms: it appears to modulate hepatic signaling cascades (e.g., GH-Jak2-STAT5-IGF1, PI3K-Akt, and Erk/MAPK pathways) and improve gut function. Similar to Scy-hepc in fish (68), it increases trypsin/amylase activity and nutrient transporters, improving absorption (69, 70). It also reduces cecal pH—indicating resistance-free antibacterial effects that enhance digestibility—and enriches Lactobacillus, Lactococcus, and Parabacteroides. These bacteria produce bacteriocins/organic acids, boosting nutrient availability and gut homeostasis. Previous antimicrobial peptide studies primarily focused on *in vitro* activity or single-parameter assessments. In contrast, this research directly compared IsCT with ciprofloxacin, a globally adopted growth-promoting antibiotic in poultry production. Critically, our results demonstrate that dietary IsCT supplementation achieves growth-promoting efficacy comparable to ciprofloxacin, with optimal effects observed at 100 mg/kg IsCT during the 22–42 day phase. This evidence-based comparison establishes a realistic foundation for IsCT’s commercial application as a viable antibiotic replacement.

### 4.4 Effects of IsCT on immune performance

The animal immune system maintains homeostasis through the regulation of inflammatory factor secretion (71). As pivotal immune organs in avian species, the liver, thymus, and bursa of Fabricius jointly sustain organismal health (72). IsCT supplementation significantly increased the bursa of Fabricius index in young broilers and elevated serum immunoglobulin (IgA, IgG, IgM) levels throughout the trial. These results indicate that IsCT not only exerts direct antibacterial effects but also modulates host immune function. The immunomodulatory mechanisms of AMPs like IsCT may include: neutralization of bacterial endotoxins such as LPS; regulation of cytokine production; enhancement of chemokine activity; and modulation of signaling pathways involved in inflammation (e.g., TLR4/NF-κB). Xie et al. (7) show 100 mg/kg Plectasin moderately improves immune organ indices and elevates immunoglobulins; Patyra et al. (6) confirmed defensins, cecropins, and moricins similarly enhance immunoglobulin levels—aligning with current findings.

AMPs can modulate the host immune response through various mechanisms. Their immunoregulatory functions include balancing the production of anti-inflammatory and pro-inflammatory cytokines, neutralizing LPS and endotoxins (73), enhancing chemokine expression, and regulating the excessive release of cytokines such as TNF-*α* and IL-1*β* (74). These actions help alleviate inflammatory responses and prevent tissue damage caused by overactivation of the immune system (75–77). For instance, human β-defensin 3 (hBD3) can bind to both LPS and TLR4, thereby blocking TLR4 activation and reducing the activity of MyD88, TRIF, and NF-κB (78). Similarly, lactoferrin binds to LPS from *Porphyromonas gingivalis* and CD14, interfering with the formation of CD14–LPS complexes and downregulating the TLR4 signaling pathway (79, 80). In a rat model, Nal-P-113 was shown to reduce the production of IL-1β and TNF-α in Pseudomonas-infected mice (81). Furthermore, AMPs can enhance the host’s antioxidant capacity and mitigate oxidative damage to immune cells. For example, Fang et al. (82) found that AMPs can improve an animal’s ability to cope with oxidative stress and increase the activity of antioxidant enzymes. As an antibacterial substance, IsCT significantly influences intestinal development and immune function through modulation of the gut microbiota. Chen et al. (83) reported that dietary supplementation with *Litsea cubeba* essential oil (LCO) improved growth performance and immune function in finishing pigs by modulating intestinal flora. Similarly, Song et al. (84) demonstrated that supplementation with soy milk fermented with Pleurotus eryngii peptides increased the abundance of beneficial gut bacteria and enhanced antioxidant capacity and immune responses in mice. Additionally, tryptophan—a constituent amino acid of IsCT—can be metabolized into bioactive compounds that modulate the TLR4 signaling pathway (85), thereby promoting the resolution of inflammation.

### 4.5 Effects of IsCT on intestinal morphology

Optimal intestinal morphology enhances nutrient absorption efficiency, with structural changes directly correlating with improved absorptive capacity (86). Increased villus height expands the absorptive surface area, thereby elevating nutrient utilization and enhancing broiler growth performance (87). IsCT supplementation significantly improves intestinal villus height, crypt depth, and the villus-height-to-crypt-depth ratio, particularly in the jejunum and ileum. These structural changes reflect enhanced absorptive capacity and overall intestinal health. The improvement in gut morphology may be attributed to a reduction in pathogenic bacteria and associated inflammation, the promotion of beneficial microbiota such as Lactobacillus and Bacteroidetes, and the upregulation of tight junction proteins and mucosal barrier function through signaling pathways such as aPKC and Rac1. The heightened responsiveness of the jejunum to IsCT treatment aligns with its primary role in nutrient absorption, whereas effects on the ileum are more closely associated with immune modulation and microbial activity. Zhu et al. (88) report that antimicrobial peptide Mastoparan X (MPX) supplementation improves villus morphology, creating a favorable microenvironment for intestinal health. Similarly, Liu et al. (87) find that the antimicrobial peptide CADN significantly increases villus height and width while enhancing structural integrity in broilers. These morphological improvements likely operate through multiple mechanisms. Alterations in the structure of the gut microbiota may represent a key underlying mechanism for this phenomenon. The gut microbiome plays a crucial role in intestinal development. Bai et al. (89) found that a hydrolyzed protein formula improved the gut microbiota, thereby enhancing intestinal development in low birth weight piglets. Similarly, Wei et al. (90) reported that sulfated fucan-induced modulation of the gut microbiota upregulates the expression of tight junction proteins in mice, leading to improved intestinal function. Wang et al. (91) observe that AMP supplementation significantly reduces aerobic bacteria while increasing beneficial genera (e.g., *Firmicutes* and *Bacteroidetes*), thereby improving the intestinal microenvironment. This microbial rebalancing mitigates intestinal inflammation and supports healthy villus development (92, 93). The antimicrobial peptide AMP-IBP5 is demonstrated to enhance barrier function in both cutaneous and intestinal tissues by activating the atypical protein kinase C (aPKC) and Rac1 signaling pathways, thereby upregulating tight junction protein expression (94).

Notably, Zhu et al.’s findings of pronounced jejunal villus height and villus height-to-crypt depth (VH/CD) ratio enhancement align with our results. This jejunal sensitivity may stem from its primary role in nutrient absorption, where morphological efficiency critically determines absorptive capacity. Conversely, AMPs may exert stronger effects on ileal morphology through immunomodulation and tissue repair mechanisms (95, 96), potentially explaining segment-specific responses.

### 4.6 Correlation analysis

In this study, significant positive correlations are observed between the villus-to-crypt ratio, immunoglobulin levels, and muscle yield in broilers at both 21 and 42 days of age. Broiler intestinal development is closely linked to growth performance, as gut health directly influences nutrient absorption, immune function, and overall growth efficiency. Building upon the significant correlations observed between gut morphology (villus-to-crypt ratio), immunity (immunoglobulin levels), and muscle yield, a potential mechanistic action of IsCT in enhancing broiler performance can be proposed. We hypothesize that IsCT primarily acts through a gut-immune axis synergy that optimizes nutrient partitioning toward muscle growth rather than fat deposition or inflammatory processes. Firstly, improved intestinal health, evidenced by the increased villus-to-crypt ratio, directly enhances nutrient absorption surface area and efficiency. This aligns with existing literature where enhanced gut morphology reduces FCR and supports growth (95). The superior nutrient availability subsequently provides more substrates for protein synthesis and muscle development (97–99). Secondly, the elevated immunoglobulin levels (IgA, IgG, IgM) indicate a potentiated humoral immune response. A robust yet balanced immune system minimizes the metabolic cost of inflammation, as chronic immune activation diverts energy and nutrients away from growth. The reduction in abdominal fat percentage observed in our study may partly result from this reallocation of energy resources. This is consistent with findings that immunomodulators (e.g., probiotics, algal extracts) can simultaneously improve immunity and growth performance (100, 101).

Based on these findings, we propose that IsCT’s antibacterial function reduces the intestinal pathogen load, thereby diminishing constant immune stimulation and gut damage. This allows for simultaneous improvement in gut morphology and a shift toward a more efficient immune profile. The concomitant enhancement in both nutrient absorption capacity and immune efficiency creates a synergistic effect, leading to the superior carcass quality (increased meat yield, reduced fat) documented in our results.

### 4.7 Future applications and research directions

IsCT demonstrates a unique dual functionality that integrates potent antibacterial activity with significant immunostimulatory effects. *In vitro* analyses confirmed its strong bactericidal effects against major poultry pathogens, with MIC values ranging from 32 to 128 μg/mL, while *in vivo* trials showed elevated immunoglobulin levels (IgA, IgG, IgM) and an enhanced bursal index. This multifunctional profile parallels the broad efficacy of traditional growth-promoting antibiotics but without conferring the same risks of drug resistance, highlighting its potential as a sustainable alternative in poultry production. When compared to well-studied AMPs such as Cecropins, Lactoferrampin-lactoferricin, and HDP-WK3, IsCT offers several distinctive advantages: its short sequence (13 amino acids) simplifies and reduces the cost of synthesis—approximately 43% that of producing longer peptides like Cecropin—and improves metabolic stability by minimizing protease-sensitive sites, thereby increasing its bioavailability in the gut (12, 102, 103). Furthermore, IsCT exhibits broad-spectrum antibacterial activity, with MIC values against *E. coli* and Salmonella generally lower than those of many reference AMPs (104), and it maintains low hemolytic activity, comparable to Cecropin and substantially lower than Melittin and LL-37, indicating a favorable safety profile for veterinary use (105–107). In broiler feeding trials, IsCT supplementation led to a 16% increase in ADG during both the starter (1–21 days) and grower (22–42 days) phases, outperforming other AMPs such as *Musca domestica* cecropin and Microcin C7, which typically improve ADG (104, 108, 109). This consistent efficacy throughout the growth cycle underscores its reliability and functional advantage. Beyond its direct antimicrobial and growth-promoting effects, IsCT shows promise in addressing antimicrobial resistance (AMR) through multi-target mechanisms including, membrane disruption and intracellular interference, which reduce the likelihood of resistance development (110, 111). Additionally, its capacity to modulate host immunity and its environmentally benign profile—degrading into natural amino acids without residue accumulation—further support its potential as part of a sustainable farming strategy (112).

Nevertheless, this study has several limitations that should be acknowledged. First, the relatively short trial duration precludes assessment of IsCT’s effects on growth performance, immune parameters, and potential long-term toxicity throughout the entire production cycle of yellow-feathered broilers. Furthermore, the experimental design may introduce selection bias; although birds were randomly allocated, the selection was from a single hatchery and a specific genetic line, which may limit the generalizability of our findings to other populations or breeds under different management conditions. Second, as a bioactive antimicrobial agent, its impact on gut microbiota composition was not investigated. This omission constrains our mechanistic understanding of how IsCT influences intestinal health and nutrient absorption, as the microbiome is a key mediator of these processes. The absence of metagenomic or 16S rRNA sequencing data represents a significant constraint on fully interpreting the gut morphology and performance results. Third, while improvements in growth and immune responses were documented, the underlying cellular and molecular mechanisms remain unexplored. Future studies should employ transcriptomic or proteomic approaches to identify key signaling pathways (e.g., NF-κB, mTOR) activated by IsCT supplementation. Finally, the exclusive focus on yellow-feathered broilers, while justified for this economically important species, limits extrapolation of the results to other livestock species such as swine or ruminants. Additionally, the chosen dose range (25–200 mg/kg), while based on previous literature, might not have captured the optimal dose for all response parameters, and the dose-interval effects warrant more detailed investigation in the future.

## 5 Conclusion

IsCT exhibits broad-spectrum antibacterial activity. Dietary supplementation with IsCT during the grower phase of broilers effectively improves growth performance, enhances immune function, and optimizes carcass quality. Comprehensive evaluation revealed that an inclusion level of 100 mg/kg IsCT is optimal. However, these findings are limited to yellow-feathered broilers under short-term feeding conditions, and the underlying mechanisms require further investigation.

## Data Availability

The raw data supporting the conclusions of this article will be made available by the authors, without undue reservation.
