# Supplementary material for: Safety and effects of scorpion-derived antimicrobial peptides as an alternative to antibiotic growth promoters in broilers: growth performance, immune function, and intestinal development
Source: Front Vet Sci. 2025 Oct 9;12:1677663. doi: 10.3389/fvets.2025.1677663 (PMC12548376; doi:10.3389/fvets.2025.1677663)
Supplement: Supplementary file 1 [file Table_1.DOCX]

**Supplementary Material**

**Table S1**. Daily Temperature, Humidity, and THI Profiles During the Broiler Rearing Period

| Experimental Day | Dry-Bulb Temperature (℃) | RH | THI |
| --- | --- | --- | --- |
| 1 | 35.2 | 64% | 75.4 |
| 2 | 35 | 68% | 75.2 |
| 3 | 35.2 | 69% | 75.4 |
| 4 | 34.8 | 62% | 75.1 |
| 5 | 34.8 | 65% | 75.1 |
| 6 | 35 | 64% | 75.2 |
| 7 | 34.8 | 64% | 75.1 |
| 8 | 32.4 | 63% | 73.1 |
| 9 | 32.2 | 63% | 72.9 |
| 10 | 32.4 | 62% | 73.1 |
| 11 | 32.2 | 62% | 72.9 |
| 12 | 32.0 | 61% | 72.8 |
| 13 | 29.0 | 65% | 70.3 |
| 14 | 28.8 | 61% | 70.2 |
| 15 | 29.0 | 62% | 70.3 |
| 16 | 29.0 | 65% | 70.3 |
| 17 | 29.0 | 65% | 70.3 |
| 18 | 29.2 | 69% | 70.5 |
| 19 | 29.0 | 61% | 70.3 |
| 20 | 29.4 | 60% | 70.6 |
| 21 | 28.8 | 60% | 70.2 |
| 22 | 26.4 | 63% | 68.2 |
| 23 | 26.2 | 62% | 68.0 |
| 24 | 26.0 | 61% | 67.9 |
| 25 | 26.0 | 68% | 67.9 |
| 26 | 25.6 | 65% | 67.5 |
| 27 | 25.6 | 65% | 67.5 |
| 28 | 25.6 | 65% | 67.5 |
| 29 | 22.8 | 65% | 65.3 |
| 30 | 22.6 | 64% | 65.1 |
| 31 | 22.8 | 62% | 65.3 |
| 32 | 22.6 | 60% | 65.1 |
| 33 | 21.6 | 64% | 64.3 |
| 34 | 22.4 | 62% | 64.9 |
| 35 | 22.6 | 61% | 65.1 |
| 36 | 21.8 | 70% | 64.4 |
| 37 | 22.6 | 68% | 65.1 |
| 38 | 22.4 | 66% | 64.9 |
| 39 | 22.6 | 70% | 65.1 |
| 40 | 22.8 | 68% | 65.3 |
| 41 | 22.8 | 69% | 65.3 |
| 42 | 21.4 | 67% | 64.1 |

**Note:**THI=(1.8×Tdb+32)-[(0.55-0.0055×RH)×(1.8×Tdb-26.8)]

Where Tdb = dry bulb temperature (°C); RH = relative humidity (%).


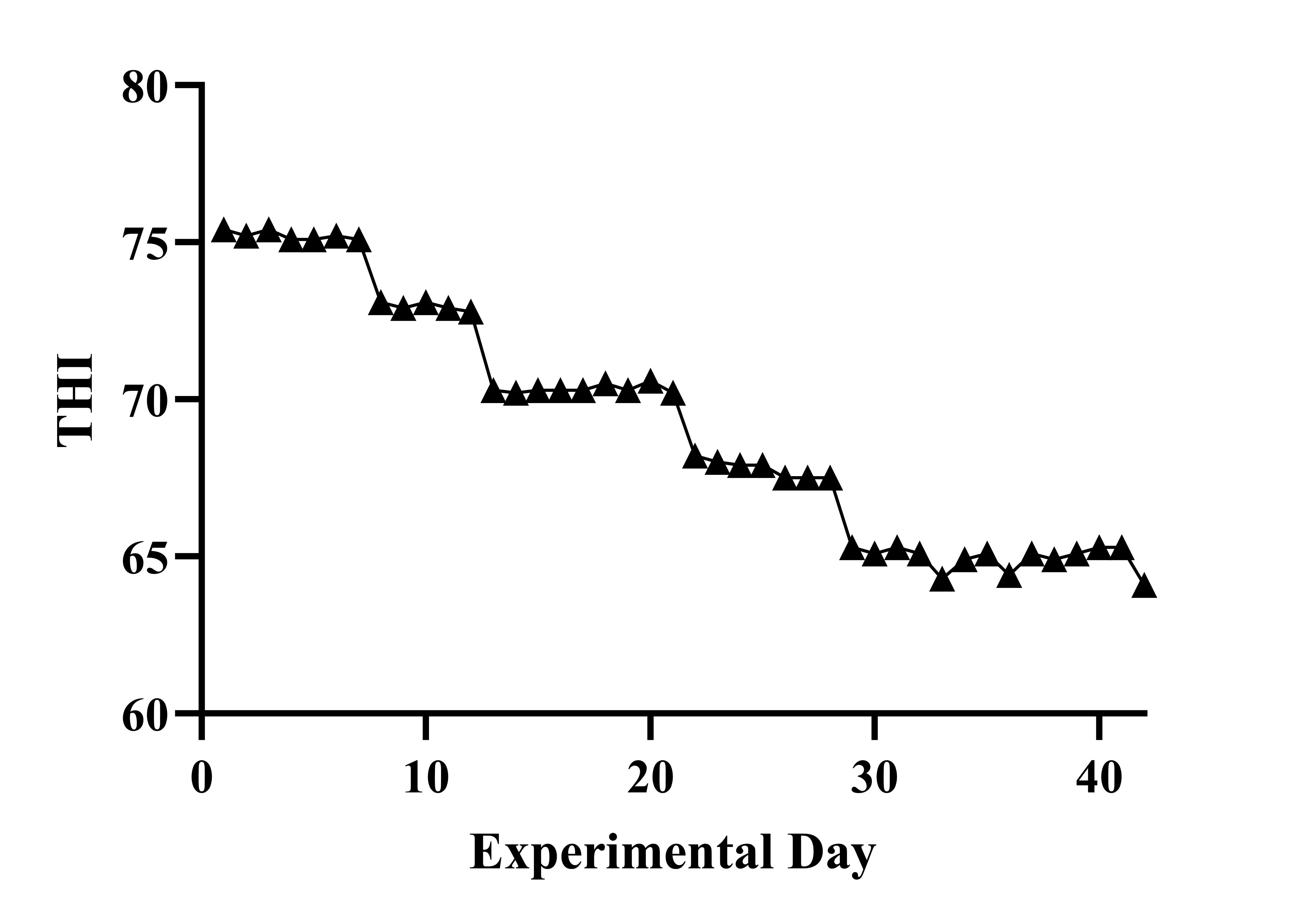


**Figure. S1.** THI During the Broiler Rearing Period
